# Meta-analysis examining overall survival in patients with pancreatic cancer treated with second-line 5-fluorouracil and oxaliplatin-based therapy after failing first-line gemcitabine-containing therapy: effect of performance status and comparison with other regimens

**DOI:** 10.1186/s12885-020-07110-x

**Published:** 2020-07-08

**Authors:** Zev A. Wainberg, Kynan Feeney, Myung Ah Lee, Andrés Muñoz, Antonio Cubillo Gracián, Sara Lonardi, Baek-Yeol Ryoo, Annie Hung, Yong Lin, Johanna Bendell, J. Randolph Hecht

**Affiliations:** 1 Department of Medicine, Division of Hematology/Oncology, David Geffen School of Medicine, University of California Los Angeles, Los Angeles, CA USA; 2 Notre Dame University, Fremantle and Edith Cowan University Joondalup, Perth, Australia; 3 Catholic University of Korea, Seoul, South Korea; 4 Hospital General Universitario Gregorio Marañón, Madrid, Spain; 5 HM Universitario Sanchinarro, Centro Integral Oncológico Clara Campal HM-CIOCC, Madrid, Spain; 6 Departamento de Ciencias Médicas Clínicas Universidad San Pablo CEU, Madrid, Spain; 7 Istituto Oncologico Veneto – IRCCS, Padova, Italy; 8 Asan Medical Center, University of Ulsan College of Medicine, Seoul, South Korea; 9 ARMO Biosciences, a wholly owned subsidiary of Eli Lilly and Company, Redwood City, CA USA; 10 Eli Lilly and Company, Indianapolis, IN USA; 11 Sarah Cannon Research Institute/Tennessee Oncology, Nashville, TN USA

**Keywords:** Pancreatic cancer, Metastatic, Performance status, FOLFOX, Meta-analysis

## Abstract

**Background:**

Pancreatic cancer has a poor prognosis and few choices of therapy. For patients with adequate performance status, FOLFIRINOX or gemcitabine plus nab-paclitaxel are preferred first-line treatment. 5-Fluorouracil (5-FU)–based therapy (e.g. FOLFIRI, OFF, or FOLFOX) are often used in patients who previously received gemcitabine-based regimens. A systematic review was conducted of the safety and efficacy of FOLFOX for metastatic pancreatic cancer following prior gemcitabine-based therapy. A Bayesian fixed-effect meta-analysis with adjustment of patient performance status (PS) was conducted to evaluate overall survival (OS) and compare outcomes with nanoliposomal irinotecan combination therapy.

**Methods:**

PubMed.gov, FDA.gov, ClinicalTrials.gov, congress abstracts, Cochrane.org library, and EMBASE database searches were conducted to identify randomized controlled trials of advanced/metastatic disease, prior gemcitabine-based therapy, and second-line treatment with 5-FU and oxaliplatin. The database search dates were January 1, 1990–June 30, 2019. Endpoints were OS and severe treatment-related adverse events (TRAEs). Trial-level PS scores were standardized by converting Karnofsky grade scores to Eastern Cooperative Oncology Group (ECOG) Grade, and overall study-weighted PS was calculated based on weighted average of all patients.

**Results:**

Of 282 studies identified, 11 randomized controlled trials (*N* = 454) were included in the meta-analysis. Baseline weighted PS scores predicted OS in 10 of the 11 studies, and calculated PS scores of 1.0 were associated with a median OS of 6.3 months (95% posterior interval, 5.4–7.4). After adjusting for baseline PS, FOLFOX had a similar treatment effect profile (median OS, range 2.6–6.7 months) as 5-FU/leucovorin plus nanoliposomal irinotecan therapy (median OS, 6.1 months; 95% confidence interval 4.8–8.9). Neutropenia and fatigue were the most commonly reported Grade 3–4 TRAEs associated with FOLFOX.

**Conclusions:**

Baseline PS is a strong prognostic factor when interpreting the efficacy of 5-FU and oxaliplatin-based therapy of pancreatic cancer after progression on first-line gemcitabine-based regimens. When baseline PS is considered, FOLFOX has a similar treatment effect as 5-FU and nanoliposomal irinotecan therapy and a comparable safety profile. These findings suggest that 5-FU and oxaliplatin-based therapies remain an acceptable and alternative second-line treatment option for patients with pancreatic cancer and adequate PS (e.g. ECOG 0–1) following gemcitabine treatment.

## Background

Pancreatic cancer is the seventh leading cause of global cancer death [1] and the third most common cause of cancer-related death in the United States [2]. It is usually diagnosed at an advanced stage, and 80–90% of patients with pancreatic cancer have unresectable tumors. For patients with metastatic disease, the 5-year survival rate is less than 10% [3]. The National Comprehensive Cancer Network (NCCN) 2019 guidelines recommend chemotherapy with FOLFIRINOX [4] or gemcitabine plus nab-paclitaxel [5] as preferred options for patients with an acceptable baseline performance status (Eastern Cooperative Oncology Group performance status [ECOG PS] score of 0–1) [6]. Cell-autonomous mechanisms of resistance to chemotherapy, however, further limit therapeutic options, and there have been multiple negative randomized trials in the adjuvant and first-line setting [7]. Immunotherapies explored so far have not demonstrated improved benefits over chemotherapy perhaps because tumor cells are nonimmunogenic in nature and are characterized by poor antigenicity [8]. Only 1% of patients with pancreatic cancer have tumors with high levels of microsatellite instability (MSI-H) or mismatch repair deficiencies (dMMR) and are considered to be candidates for checkpoint inhibitors [9, 10]. Furthermore, in the small minority of patients with pancreatic cancer who have germline *BRCA* mutations (4–7%), progression-free survival (PFS) following poly(adenosine diphosphate–ribose) polymerase (PARP) inhibitor therapy was not influenced by prior response to platinum-based therapy [11].

In general, most guidelines recommend the use of gemcitabine as monotherapy or as part of a combination therapy regimen for patients previously treated with FOLFIRINOX or other fluoropyrimidine-based therapy [6]. For patients previously treated with gemcitabine-based regimens, 5-FU–based therapy including FOLFIRI, OFF, and FOLFOX has been recommended [6]. Recently, the Food and Drug Administration (FDA) approved nanoliposomal irinotecan in combination with 5-FU and leucovorin as second-line therapy after previous gemcitabine-based therapy (NAPOLI-1) [12]. Based on the findings from the NAPOLI-1 study, updated guidelines recommend the use of nanoliposomal irinotecan with fluorouracil and leucovorin in patients with metastatic pancreatic cancer after prior gemcitabine-based therapy [13]. In the NAPOLI-1 study, the median overall survival (OS) was 6.1 months (95% confidence interval [CI] 4.8–8.9) for the combination of nanoliposomal irinotecan/5-FU/leucovorin compared with 4.2 months (95% CI 3.3–5.3) for 5-FU/leucovorin alone with a hazard ratio of 0.67 (95% CI 0.49–0.92; *P* = .012) in patients with Karnofsky PS scores of 70 and above [12]. Survival benefits of this regimen were numerically similar to historically 5-FU–based therapy. For example, the phase III CONKO-003 trial of OFF demonstrated a median OS of 5.9 months [14]. More recently, a randomized phase II trial of mFOLFOX reported a median OS of 6.7 months in patients previously treated with gemcitabine [15], and despite not meeting its primary endpoint, the phase III PANCREOX study of mFOLFOX demonstrated a median OS of 6.1 months [16].

In the past, many prognostic factors have been identified and considered, such as hemoglobin level, tumor burden, liver metastases, venous thromboembolism, baseline expression of B7H1 or B7H4, and baseline CA19–9 [17–23]. One of the most significant prognostic factors is baseline ECOG PS. For example, one small, single-arm, phase II cohort study demonstrated a median OS for second-line FOLFOX with a median survival of 4.3 months. When patients were stratified by baseline ECOG PS, the median OS was 5.9 months for patients with adequate PS (i.e., ECOG PS scores, 0–1) and 2.6 months for those with ECOG PS scores ≥2 [24]. In this paper, we performed a systematic review to better characterize the safety and efficacy of FOLFOX treatment for patients with metastatic pancreatic cancer following prior gemcitabine-based therapy. A Bayesian meta-analysis with adjustment of patient PS was conducted to evaluate the median OS and cross-compare with nanoliposomal irinotecan combination therapy.

## Methods

### Literature search

Studies were identified from searches conducted in PubMed.gov, FDA.gov, ClinicalTrials.gov, abstracts from individual congress proceedings, the Cochrane.org library, and the EMBASE database between January 1, 1990 and June 30, 2019. The search terms used were “pancreatic cancer”, “gemcitabine”, “FOLFOX”, 5-fluorouracil”, “oxaliplatin”, and “leucovorin”.

### Inclusion and exclusion criteria

Trials meeting the following criteria were included in the meta-analysis: 1) patients with locally advanced and metastatic disease, 2) patients who received prior gemcitabine-containing treatment, 3) second-line treatment regimens included 5-FU and oxaliplatin, and 4) reported data included median OS, severe (Grades 3–4) treatment-related adverse events (TRAEs), based on the Common Terminology Criteria for Adverse Events (CTCAE) v4.0 [25]. Trials meeting the following criteria were excluded from the meta-analysis: 1) patients who received prior treatment with 5-FU and oxaliplatin for locally advanced or metastatic pancreatic cancer, 2) patients who received an oral fluoropyrimidine, or irinotecan, capecitabine, or cisplatin as second-line treatment, and 3) patient PS was not reported.

### Data collection and analysis

Two reviewers independently evaluated the literature identified from the database searches. For studies reported in different publications, the most recent study was retained, and the other version was excluded. The information extracted from each study included author names, publication year, number of patients, number of survival events, median OS, and severe adverse events. Any discrepancies in study eligibility or data extraction were reconciled. Studies were excluded if the full text of the publication was not available or if PS or median OS data were not reported.

### Statistical analyses

The primary endpoint and secondary endpoints were median OS and severe TRAEs for patients who received FOLFOX or 5-FU/oxaliplatin–based therapy following prior gemcitabine–based regimens for metastatic pancreatic cancer. Adjusted PS was included in the meta-analysis model as follows. To standardize the trial-level PS, Karnofsky grade was converted to ECOG Grade according to Oken et al. [26], and the overall study-weighted PS was calculated based on the weighted average. For example, ECOG 0–1 was converted to numerical value 1, and ECOG 2, 3, and 4 were converted to numerical values 2, 3, and 4, respectively. For a study with *w*_1_% of the patients had ECOG 0–1 and *w*_2_% with ECOG 2. The weighted trial performance was calculated as: 1× *w*_1_% + 2× *w*_2_%. A Bayesian fixed-effect meta-analysis was performed for the median OS with weighted trial PS as a predictor. A noninformative prior was used to establish the relationship between log transformation of median OS and PS. The noninformative prior was assumed for the related parameters. The posterior median of OS and 95% posterior interval (PI) were summarized for patients with ECOG PS ≤1. For safety, Grade 3/4 clinically relevant toxicities that were reported in ≥10% of patients in any trial were pooled together to evaluate the toxicity of the treatment regimen. To be conservative, trials that did not report a specific adverse event were removed from the group of evaluable patients. All analyses were performed in R 3.5.0.

## Results

### Study selection

**Fig. 1:**
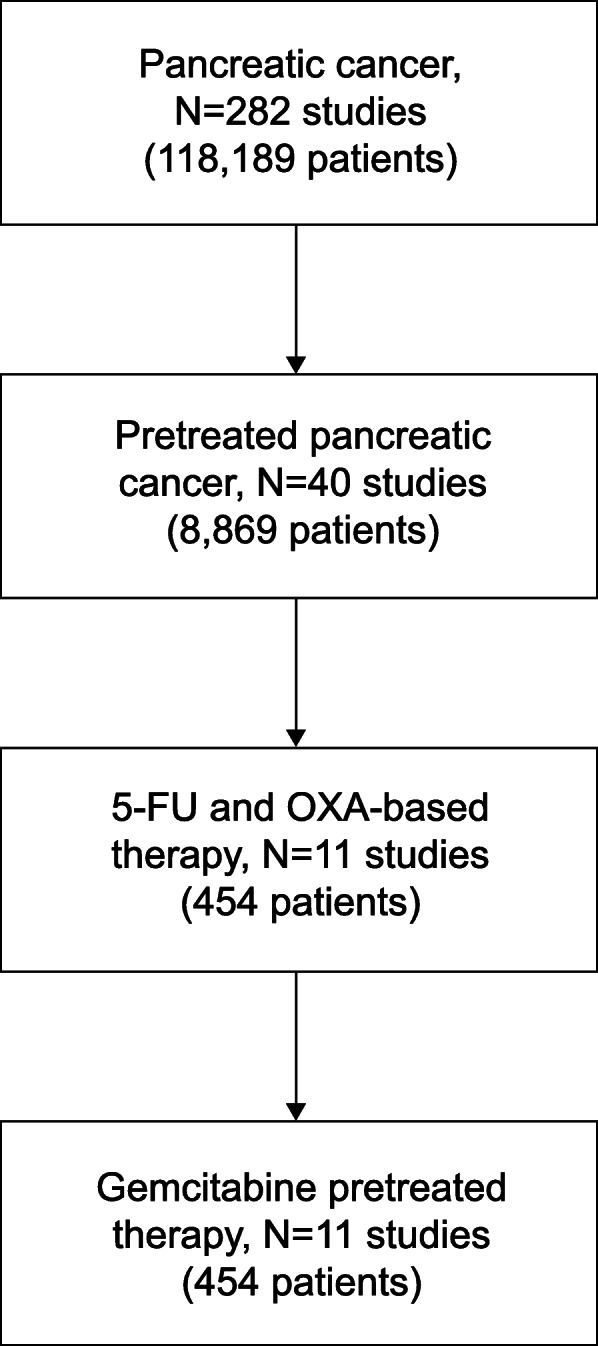
CONSORT diagram

The CONSORT flow chart that illustrates study identification and selection for the meta-analysis is shown in Fig. 1. Of 282 studies identified in the database searches, 11 were chosen for meta-analysis [14–16, 24, 27–33], and 242 studies were excluded. In total, 454 patients with pancreatic cancer were included in this meta-analysis. The 11 selected studies evaluated 5-FU and oxaliplatin-based regimens, including OFF, FOLFOX, and modified FOLFOX (mFOLFOX6, mFOLFOX4) (Table 1).

**Table 1:** Summary of 5-FU and oxaliplatin-based therapy as second-line therapy

Treatment	Author/year	*N*	Weighted PS	Original PS	Prior surgery (%)	Deaths	Median OS (m)	ORR (%)
OFF	Pelzer 2009 [28]	37	1.5	KS: Median: 70, range: [60, 90]	43	33	5.1	6
OFF	Pelzer 2011 [27]	23	1.3	KS: Median: 80, range: [70, 100]	NR	18	4.8	0
OFF	Oettle 2014 [14]	76	1.2	KS: (90–100) (53.9%), 70–80 (46.1%)	45	73	5.9	17
5-FU/OXA-based	Tsavaris 2005 [29]	30	1.7	KS: (80–100) (33.4%), 70–50 (66.7%)	NR	20	5.7	23
FOLFOX	Gebbia 2007 [30]	42	1.4	ECOG: 1 (62%), 2 (38%)	9	38	6.7	14
FOLFOX	Yoo 2009 [31]	30	1.0	ECOG: 0–1 (97%)	32	25	3.8	7
FOLFOX	Zaanan 2014 [24]	12	1.0	ECOG: 0–1 (100%)	0	10	5.9	0
FOLFOX	Zaanan 2014 [24]	12	2.5	ECOG: 2–3 (100%)	0	12	2.6	0
FOLFOX	Gill 2016 [16]	54	1.1	ECOG: 0–1 (89%), 2 (11%)	NR	47	6.1	13
FOLFOX	Berk 2012 [32]	46	1.2	ECOG: 0–1 (78%), 2 (22%)	NR	33.6	6.2	17
OFF	El-Hadaad 2013 [33]	30	1.2	ECOG: 0–1 (83.4%), 2 (16%)	NR	29	5.1	7
FOLFOX	Chung 2017 [15]	62	1.0	ECOG: 0–1 (100%)	NR	53	6.7	11

Abbreviations: *5-FU* 5-fluorouracil, *ECOG* Eastern Cooperative Oncology Group, *FOLFOX* leucovorin/5-fluorouracil/oxaliplatin, *m* months, *KS* Karnofsky status, *NR* not reported, *OFF* oxaliplatin/5-fluorouracil/leucovorin, *ORR* overall response rate, *OS* overall survival, *OXA* oxaliplatin, *PS* performance score

### Patient population

In the 454 evaluable patients, the reported PS ranged from Karnofsky performance index scores of 60–100 and ECOG PS scale scores of 0–3 (Table 1). Of 11 studies, five reported the surgical histories of the patient sample. Rates of prior surgery were 8% [34], 9% [30], 32% [31], 43% [28], and 45% [14]. The median OS ranged from 2.6 months to 6.7 months, and the overall response rate ranged from 0 to 23% (Table 1).

### Overall survival

**Fig. 2:**
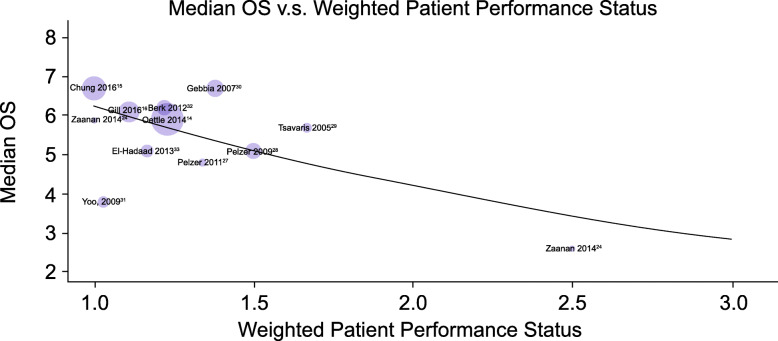
Association between median overall survival (OS) and patient performance status

**Fig. 3:**
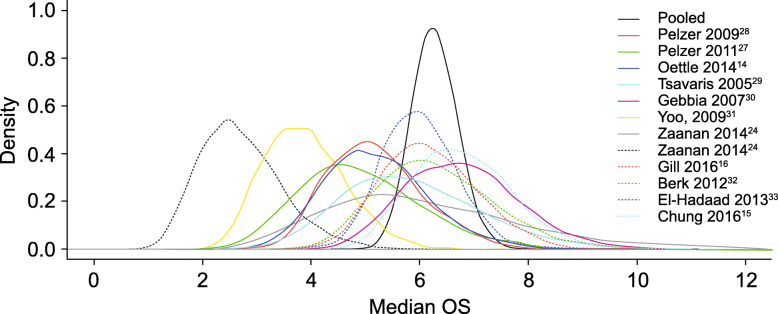
Overall survival (OS) meta-analysis of 5-fluorouracil (5-FU) and oxaliplatin (OXA)-based therapy

**Fig. 4:**
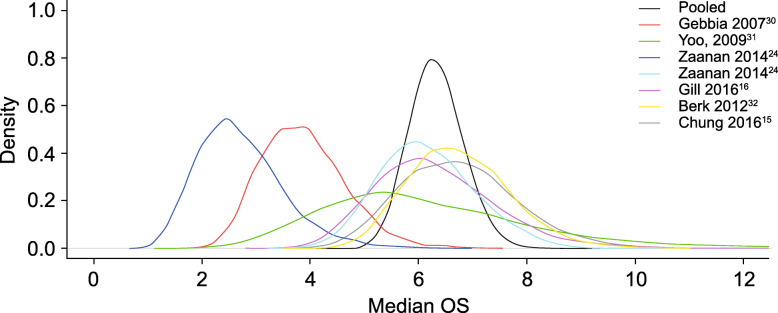
Overall survival (OS) meta-analysis of FOLFOX

Baseline weighted PS scores predicted OS in 10 of the 11 studies (Fig. 2). Results from one study were identified as an outlier, with a median OS of approximately 4 months in patients with a baseline weighted PS score of 1.0 [31]. Likely the variability was because of a long period of time between the conclusion of gemcitabine-based therapy to FOLFOX treatment (median 15 weeks, range 7.0–32.6 weeks). To maintain integrity of the analysis, the outlier was not removed from the model. Based on the Bayesian meta-analysis with the adjustment of baseline PS, for 5-FU and oxaliplatin-based therapy (Fig. 3), the median OS was 6.2 months (95% PI 5.4–7.1). For the analysis of FOLFOX therapy (Fig. 4), the median OS was 6.3 months (95% PI 5.4–7.4).

### Safety of FOLFOX

The clinically relevant Grade 3–4 TRAEs for the selected studies were pooled, and the results are summarized in Table 2. The most commonly reported Grade 3–4 TRAEs associated with FOLFOX therapy were neutropenia (21.5%) and fatigue (11.7%). Other Grade 3–4 TRAEs occurring in > 10% in any trial were neurotoxicity (5.3%), thrombocytopenia (4.9%), anemia (4.5%), diarrhea (4.2%), and vomiting (4.1%).

**Table 2:** Summary of safety for 5-FU and oxaliplatin-based therapy

Treatment	Author/year	*N*	Grade 3–4 clinically relevant toxicities > 10% in any trial						
			Diarrhea	Neutropenia	Anemia	Neurotoxicity	Fatigue^a^	Vomiting	Thrombocytopenia
OFF	Pelzer 2009 [28]	37	8.1	NR	NR	10.8	NR	13.5	0
OFF	Pelzer 2011 [27]	23	8.7	NR	NR	NR	NR	NR	NR
OFF	Oettle 2014 [14]	76	1.3	NR	3.9	NR	NR	1.3	1.3
5-FU/OXA-based	Tsavaris 2005 [29]	30	14.2	NR	3.2	4.2	0	0	3.2
FOLFOX	Gebbia 2007 [30]	42	NR	17	14	12	NR	NR	7
FOLFOX	Yoo 2009 [31]	30	0	20	3	0	14	10	3
FOLFOX	Zaanan 2014 [24]	27	0	7.4	7.4	7.4	14.8	0	11.1
FOLFOX	Gill 2016 [16]	49	2	32.7	2	4.1	14.2	4.1	8.2
FOLFOX	Berk 2012 [32]	46	2	22	0	NR	NR	2	7
OFF	El-Hadaad 2013 [33]	30	3.3	23.2	6.6	6.6	NR	3.3	6.6
FOLFOX	Chung 2017 [15]	62	6.5	NR	3.2	0	12.9	4.8	NR
Evaluable patients for each AE, *n*			410	224	392	307	198	387	367
Weighted average (%)			4.2	21.5	4.5	5.3	11.7	4.1	4.9

Abbreviations: *5-FU* 5-fluorouracil, *AE* adverse event, *FOLFOX* leucovorin/5-fluorouracil/oxaliplatin, *N* patients in each study, *n* evaluable patients for each AE, *NR* not reported, *OFF* oxaliplatin/5-fluorouracil/leucovorin, *OXA* oxaliplatin

^a^Fatigue includes reported terms of fatigue and asthenia

## Discussion

The prognosis of pancreatic cancer remains dismal, and the primary first-line treatments for patients with metastatic disease are gemcitabine-based combinations and FOLFIRINOX. For patients previously treated with gemcitabine, second-line 5-FU–based therapy including FOLFIRI, FOLFOX, and OFF have been recommended [6]. In randomized trials, oxaliplatin–based regimens in the second-line setting, such as CONKO-003 and PANCREOX, have had conflicting efficacy results [35]. In the CONKO-003 trial, the OFF regimen was superior to FF (leucovorin and 5-FU) with a median OS of 5.9 vs. 3.3 months, respectively [14]. On the other hand, the PANCREOX study compared a different oxaliplatin, 5-FU, and leucovorin-containing regimen (mFOLFOX6) with 5-FU/LV, with a median OS of 6.1 vs. 9.9 months, respectively [16]. While mFOLFOX6 produced results consistent with prior studies of oxaliplatin and 5-FU combinations, the 5-FU/LV control arm demonstrated surprisingly prolonged survival. One factor that may have contributed to these findings was an imbalance in several baseline characteristics. For example, the median time from diagnosis of advanced disease to treatment was longer in the mFOLFOX6 arm compared with the 5-FU/LV arm (7.9 vs. 5.7 months, respectively), and a higher proportion of patients in the mFOLFOX6 arm than in the 5-FU/LV arm had baseline ECOG PS scores of 2 (11.1% vs. 5.7%). Additionally, fewer patients in the mFOLFOX6 arm than the 5-FU/LV arm received post-discontinuation therapy (7% vs. 23%, respectively). It is important to remember that these are relatively small studies of fewer than 200 patients each, and comparisons are fraught because of inherent methodologic differences.

The systematic literature review and meta-analysis reported here was conducted in an attempt to overcome the variability induced by small sample sizes. In addition, after adjusting for PS, the meta-analysis of 5-FU and oxaliplatin-based therapy (e.g., FOLFOX) demonstrated a numerically similar treatment effect (median OS range 2.6–6.7 months; Table 1) compared with 5-FU/LV plus nanoliposomal irinotecan combination therapy in the NAPOLI-1 trial (median OS 6.1 months; 95% CI 4.8–8.9) (Table 3) [12]. For patients with ECOG PS of 0 or 1, the median OS was 6.2 months (95% PI 5.4–7.1) for patients who received the oxaliplatin, 5-FU, and LV regimen. In addition, for the subset meta-analysis of FOLFOX therapy (Fig. 4), the median OS demonstrated consistent results with median OS of 6.3 months (95% PI 5.4–7.4). The most commonly reported Grade 3–4 TRAEs associated with FOLFOX therapy were neutropenia (21.5%) and fatigue (11.7%). Other Grade 3–4 TRAEs occurring in > 10% in any trial were neurotoxicity (5.3%), thrombocytopenia (4.9%), anemia (4.5%), diarrhea (4.2%), and vomiting (4.1%) (Table 2). Based on an indirect comparison, this adverse event profile was similar to the findings of the NAPOLI-1 trial (Table 4).

**Table 3:** Baseline and efficacy profile for nanoliposomal irinotecan-based therapy from NAPOLI-1 [12]

Characteristics^a^	Results
*N*	117
Karnofsky performance 100–80	91%
Lines of prior therapy: 0/1/2+ (%)	13/53/34
Prior therapy: Gemcitabine mono/combination/5-FU-based (%)	45/55/43
Median OS (95% CI)	6.1 months (4.8–8.9)

Abbreviations: *5-FU* 5-fluorouracil, *CI* confidence interval, *ECOG* Eastern Cooperative Oncology Group, *N* patients in study, *OS* overall survival

^a^For patients with ECOG 0–1, the poster median of the median OS for 5-FU and oxaliplatin-based therapy and FOLFOX in second-line are 6.2 months and 6.3 months, respectively

**Table 4:** Safety profile for nanoliposomal irinotecan-based therapy [12]

Grade 3–4 AEs	Nanoliposomal irinotecan-based therapy (%)	5-FU and OXA-based therapy weighted average (%) [range]
Diarrhea	13	4.2 [0, 14.2]
Vomiting	11	4.1 [0, 13.5]
Fatigue	14	11.7 [0, 14.8]
Neutropenia	27	21.5 [7.4, 32.7]
Anemia	9	4.5 [0, 14]
Hypokalemia	3	NR
Neurotoxicity	NR	5.3 [0, 12]
Thrombocytopenia	NR	4.9 [0, 11.1]

Abbreviations: *5-FU* 5-fluorouracil, *AE* adverse event, *NR* not recorded, *OXA* oxaliplatin

These analyses are not without limitations. Our ability to adjust survival outcomes for other potential prognostic factors was hindered because we did not have access to the full study datasets. For example, prior surgery, levels of the CA-19-9 antigen, baseline hemoglobin levels, *BRCA1* or *BRCA2* mutation status, or the time from diagnosis to the initiation of treatment were not always reported. In addition, the cross-trial comparison between the meta-analysis of the FOLFOX treatment regimen and the results from NAPOLI-1 are indirect and must be interpreted with caution.

## Conclusions

In this meta-analysis, we confirmed that baseline PS is a strong prognostic factor when interpreting the efficacy of 5-FU and oxaliplatin-based therapy after progression of pancreatic cancer with first-line gemcitabine-containing therapies. After adjusting for patient PS, the meta-analysis of 5-FU and oxaliplatin-based therapy (e.g., FOLFOX) shows a numerically similar treatment effect as 5-FU and nanoliposomal irinotecan therapy in the NAPOLI-1 trial. In addition, the adverse event profile is also comparable between the two treatment regimens. The findings from our analyses suggest that the combination of 5-FU and oxaliplatin-based therapies remains an acceptable and alternative second-line treatment option for patients with pancreatic cancer and adequate PS (e.g., ECOG 0/1) who have received gemcitabine-based therapies.

## Data Availability

All data generated or analyzed during this study are available from the publications cited in the reference list.

## Abbreviations

CI: Confidence interval
CTCAE: Common Terminology Criteria for Adverse Events
dMMR: Mismatch repair deficiencies
ECOG: Eastern Cooperative Oncology Group
FDA: Food and Drug Administration
5-FU: 5-fluorouracil
MSI-H: High level of microsatellite instability
NCCN: National Comprehensive Cancer Network
OS: Overall survival
PARP: Poly(adenosine diphosphate–ribose) polymerase
PFS: Progression-free survival
PI: Posterior interval
PS: Performance status
TRAE: Treatment-related adverse event
